# Widespread Report of Multiple Insecticide Resistance in *Anopheles gambiae* s.l. Mosquitoes in Eight Communities in Southern Gombe, North-Eastern Nigeria

**Published:** 2019-03-30

**Authors:** Adedayo Olatunbosun-Oduola, Ezra Abba, Olukayode Adelaja, Adeolu Taiwo-Ande, Kennedy Poloma-Yoriyo, Taiwo Samson-Awolola

**Affiliations:** 1 Department of Zoology, University of Ilorin, Ilorin, Kwara State, Nigeria; 2 Department of Biological Sciences, Faculty of Science, Gombe State University PMB 127, Gombe, Nigeria; 3 Public Health Division and Epidemiology, Nigerian Institute of Medical Research, Yaba, Lagos, Nigeria

**Keywords:** Multiple, Insecticide resistance, *Anopheles gambiae*, Gombe south, Nigeria

## Abstract

**Background:**

Timely entomological and insecticide resistance monitoring is a key to generating relevant data for vector management. We investigated the insecticide susceptibility status of *Anopheles gambiae* s.l. in eight rural farming communities in Southern Gombe, Nigeria.

**Methods:**

Overall, 3–5 days-old adult female *Anopheles* mosquitoes reared from field-collected immature stages between September and November, 2014 were exposed to the diagnostic doses of pyrethroids, organophosphate and carbamate insecticides using the Center for Disease Control Bottle bioassay. The observatory knockdown time from exposure to each insecticide was recorded up to two hours. The dead mosquitoes were then identified morphologically and by molecular assays.

**Results:**

Mortality results showed resistance in *An. gambiae* s.l. populations to bendiocarb (2.3–100%), deltamethrin (39–70%), pirimiphos-methyl (65–95%), dichloro-diphenyl-trichloroethane (0–38.1%), permethrin (0–46.3%) and lambda-cyhalothrin (42.5–86.4%). The few cases of full susceptibility were observed from lamdacyhalothrin exposed population of *An. gambiae* s.l. in Banbam and Pantami respectively. *An. gambiae* 177 (45%) was significantly higher (P< 0.05) than *An. arabiensis* 64 (16.3%), *An. coluzzii* 34 (8.7%) and *An. gambiae/An. coluzzii* hybrid 78 (19.8%).

**Conclusion:**

A strong evidence of widespread resistance in the major malaria vector species in Southern Gombe to all common classes of insecticides is a justification for the State Malaria Elimination Programme to consciously consider incorporating insecticide resistance management strategies into control programs in order to sustain the future of current control interventions.

## Introduction

Malaria is a major public health burden in Nigeria with over 90% of her 167 million people at constant risk. Nigeria accounts for 29% of the global malaria burden and together with the Democratic Republic of Congo contribute up to 40% of the global burden for malaria (1). *Anopheles gambiae* s.l. have been reported to be the most widespread mosquitoes responsible for malaria transmission in all the ecological zones (1). Controlling these vectors pose major challenges as the *An. gambiae* s.l. mosquito belongs to a complex with each playing different role in transmission and exhibiting varying insecticide susceptibility status across the several regions in Nigeria.

In Nigeria, vector control strategy remains one of the frontline and effective tools for controlling malaria and other insect-borne diseases. The strategy relies heavily on the use of insecticides from just four available classes of insecticides. In the face of increasing reports of insecticide resistance, sustained efficacy of these chemicals is desired. Resistance to at least one or more classes of insecticide have assumed a geographical scale in more than 50 countries (2). This is a major threat to pyre-throids which is the only class of insecticide approved for use in long lasting insecticide nets) (LLINs as compared to Indoor Residual Spray (IRS) which utilizes all the four classes (3). The delicate gains made in the global reductions of malaria deaths since 2010 due to increased funding and concerted efforts to attain control is threatened by emerging resistance to insecticides in *Anopheles* mosquitoes (2).

Insecticide resistance data exist in Nigeria; however, most of these data are not consistently monitored for all communities in Nigeria. Despite an increase in the reporting of insecticide resistance in southern (4–9) and Northern Nigeria (10–11), only very little information on insecticide resistance exist for Gombe state. This suggests a major setback for vector control and weak existing capacity for monitoring insecticide resistance. Where these are available, they are not consistently generated to guide implementation of vector control. Similarly, there is also limited information on the insecticide susceptibility status of members of the major malaria vector species: *An. gambiae* s.l. to available classes of insecticides used in malaria vector control in the Southern Gombe State. This study therefore provides information meeting the demand of The Nigerian National Malaria Strategic plan to generate surveillance data that can be used to inform policy.

This study for the first time provides such information on the susceptibility status and identity of the major malaria vectors in southern Gombe to different available classes of insecticides used in vector control.

## Materials and Methods

### Study Area

**Fig. 1.**
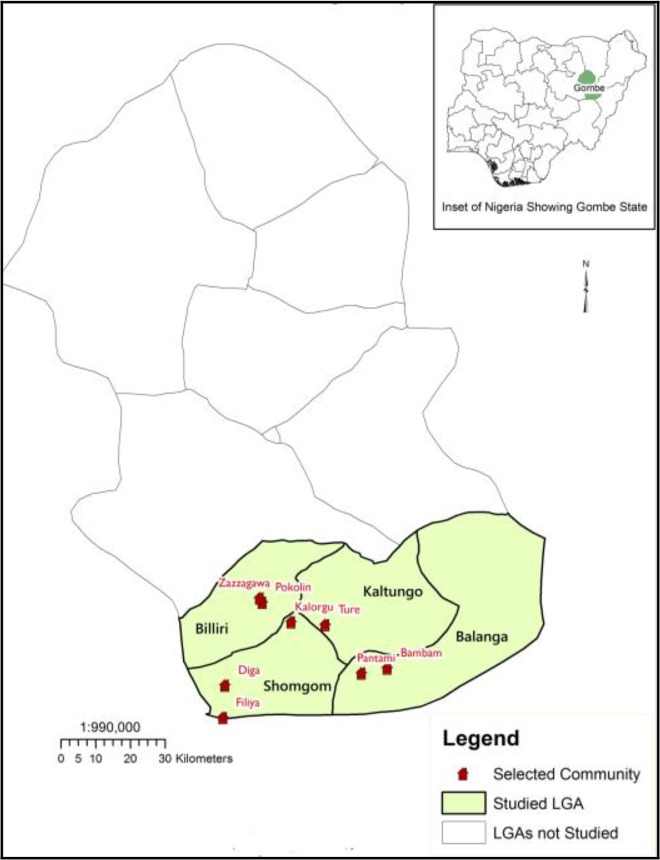
Map of Gombe South, Nigeria showing the study area and sampling localities

The study was carried out in the four Local Government Areas of southern part of Gombe state, 10°17′N, 11°10′E. Gombe is a state in North-Eastern Nigeria, with its capital at Gombe. The state has an area of 20,265km^2^ and a population of around 1.8 million. The state is characterized by two distinct seasons, which are dry season (Nov to Mar) and wet season (Apr to Oct). The vegetation of Gombe state can be described as Sudan Savanna with open grassland which dries up during the dry season. Gombe State shares boundaries with Yobe State to the North, Adamawa and Taraba States to the South, Borno State to the East, and Bauchi State to the West. The people of Gombe south are mainly farmers. They produce both food and cash crops. Among its food crops are yam, cassava, maize, millet, sorghum, cowpea, tomato, groundnut, while cotton are produced for cash. Indigenes also keep cattle, goats, sheep, horses, and donkeys and practice the traditional crafts of weaving and dyeing cotton The study was carried out in 8 farming rural communities namely: Kalorgu (N09°49′34.9″, E01117′27.1″), Ture (N09° 49′09.8″, E011° 22′44.2″), Bambam (N09° 42′22.7″, E011°32′23.5″), Pantami (N09° 41′35.4″, E011°28′23.4″), Pokolin (N09°52′39.8″, E011°12′57.5″), Zazzagawa (N09°53′20.1″, E011°12′34.7″), Filiya (N09°34′38.5″, E011°06′52.4″), Diga (N09°39′41.5″, E11°07′10.5″) (Fig. 1).

### Collection of *Anopheles* Larvae

Third to fourth instar larvae and pupae of *Anopheles* mosquito larvae were collected from breeding sites in the eight communities from four Local Government Areas (Billiri, Kaltungo, Balanga and Shomgom) of Gombe South, Nigeria. Anopheline larvae were collected from their natural breeding sites (rice farms, small pools, puddles, and potholes) using the dipping method during the months of September to Nov 2014. Coordinates of the study sites were established using The Global Positioning System (GPS).

The immature Anopheles mosquitoes collected were transported to the Gombe state malaria control insectary where they were reared to adults. The emerging adult mosquitoes were placed in the adult mosquito cages and fed with 10% sugar solution soaked in cotton wool.

### Separation of Female *Anopheles* Mosquitoes and Morphological Identification of the Mosquitoes

The adult female *Anopheles* used for the test were separated from the males. Using morphological characters (12–13) the adult female mosquitoes were identified under a dissecting microscope. The identification focused on dark spots at the upper margins of the wings which is common to all *Anopheles*. The palpis are elongated and segmented into three. Speckles on the legs, third pre-apical dark area on vein 1 with a pale interruption and tersi 1–4 with conspicuous pale bands are morphological features for the identification of *An. gambiae.*

### CDC Bottle Bioassay Method

Ten to fifteen unfed female *Anopheles* mosquitoes of 3–5 days old were introduced into four 250ml Wheaton bottles coated with Technical grade insecticide and one control bottle coated with acetone. These were provided by Center for Disease Control (CDC), Atlanta Georgia as described by the Guideline for Evaluating Insecticide Resistance in Vectors Using the CDC Bottle Bioassay). The numbers of dead and live mosquitoes were monitored at different time intervals (0, 15, 30, 35, 40, 45, 60, 75, 90, 105, 120min). This allowed the determination of the total percent mortality against time for all replicates. The tested mosquito samples were stored in eppendorf tubes containing silica gel for further analysis.

### PCR Identification of Members of the *Anopheles gambiae* complex

Mosquitoes identified as *An. gambiae* complex was subjected to species-specific polymerase chain reaction assays to identify members of the members. The molecular identification method was based on specific DNA nucleotide sequences in the intergenic spaces of the ribosomal DNA (14). Further analysis to identify the *An. gambiae* (formerly ‘S’ form) and *An. coluzzii* (formerly ‘M’ form) was determined by incubating amplified material with HhaI restriction enzymes at 37 degrees over a period of 3h to detect Restriction Fragment Length Polymorphisms (RFLPs). The enzyme HhaI produced patterns of DNA bands which differentiated *An. gambiae* from *An. coluzzii* (15).

### Data Analysis

The percentage mortality of the mosquitoes exposed to each of the insecticides was calculated as the proportion of mosquitoes that died at the diagnostic time for each of the insecticides. Correction with Abbott’s formula was not necessary as control mortalities was less than 5% throughout the test. WHO recommendations for assessing the significance of detected resistance was used. ccording to the criteria, ≥98% mortality at the recommended diagnostic time indicates susceptibility, ≤97% mortality indicates resistance (16). Data were entered into SPSS ver. 17 software (Chicago, IL, USA) and species composition in the study communities were analyzed for significant differences using analysis of variance. A general linear model procedure (t-test independent means at P< 0.05 significance) was also used to compare mortality in *A gambiae* populations exposed to two different insecticides within the same (deltamethrin/ lambda-cyhalothrin, deltamethrin/ permethrin, and lamdacyhalothrin/ permethrin) and different classes (pyrethroids and DDT, Organophosphate and Carbamate) but having the same target site.

### Ethical Approval

This study received ethical approval from University of Ilorin Ethical Research Committee.

## Results

### Insecticide susceptibility tests

**Fig. 2.**
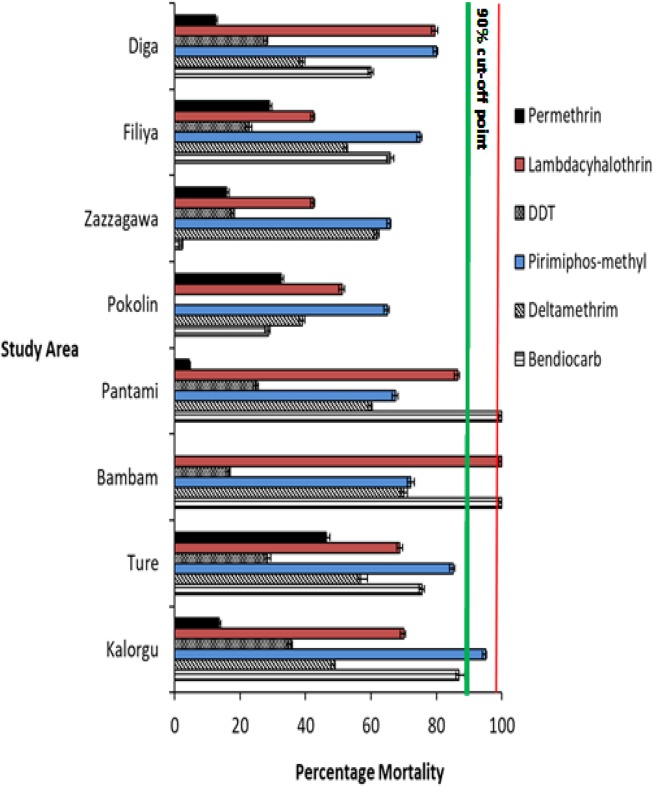
Insecticide susceptibility/resistance profile of *Anopheles gambiae* s.l. mosquitoes in eight selected communities in Southern Gombe Nigeria. Error bars represent variability in the data All charts below the red line indicate that population of *An. gambiae* in the study communities that are resistant to the classes of insecticides

Three batches of adult female Anopheles mosquitoes each containing 331, 333 and 345 *An. gambiae* s.l. mosquitoes were exposed to deltamethrin, lambda cyhalothrin and permethrin insecticides (Table 1). There was resistance (0–86.4%) in all the study communities to the three different pyrethroids tested except in Bambam community where full susceptibility (100%) of the *An. gambiae* s.l. to lamdacyhalthrin was observed (Table 1). In the same manner another batch of 342, 324 and 344 *Anopheles* mosquitoes exposed to bendiocarb, pirimiphos-methyl and DDT showed resistance (0–95%) in all the study communities (Table 2). Except in Bambam and Pantanmi where full susceptibility (100%) to bendiocarb was observed (Fig. 2). The resistance profile of the *An. gambiae* population showed that susceptibility (percentage mortality ≥97%) were found only in Bambam and Pantanmi communities.

**Fig. 3.**
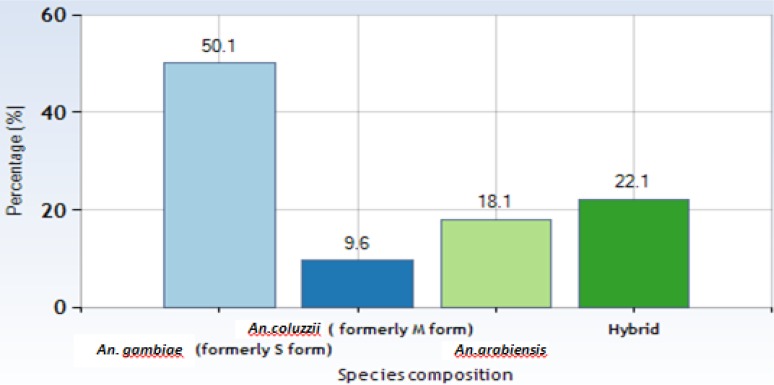
Composition of members of the *Anopheles gambiae* s.l. and hybrid (*A gambiae*/ *An. coluzzii* species in Southern Gombe

**Table 1.** Susceptibility status of *Anopheles gambiae* s.l. mosquitoes to pyrethroids insecticides

**Study Sites**	**Deltamethrin**			**Lambda-cyhalothrin**			**Permethrin**		

	**Number Assayed (331)**	**Mortality N (%)**	**Status**	**Number Assayed (333)**	**Mortality N (%)**	**Status**	**Number Assayed (345)**	**Mortality N (%)**	**Status**
**Kalorgu**	43	21 (48.8)	R	41	25 (61)	R	44	6 (13.6)	R
**Ture**	44	25 (56.8)	R	43	30 (70)	R	41	19 (46.3)	R
**Bambam**	40	28 (70)	R	40	40 (100)	S	47	0 (0)	R
**Pantami**	40	24 (58.5)	R	44	38 (86.4)	R	44	2 (4.5)	R
**Pokolin**	41	16 (39)	R	41	21 (51.2)	R	40	13 (32.5)	R
**Zazzagawa**	42	26 (61.9)	R	40	17 (42.5)	R	44	7 (15.5)	R
**Filiya**	40	21 (52.5)	R	40	17 (42.5)	R	45	13 (28.9)	R
**Diga**	41	16 (39)	R	44	35 (79.5)	R	40	5 (12.5)	R

N= number of mortality, R=Resistant, S=Susceptible, Numbers in brackets represent percentages No mortality was observed in the control experiment set up for the insecticides in each study sit

**Table 2.** Susceptibility status of *Anopheles gambiae* s.l. mosquitoes to bendiocarb, pirimiphos-methyl and DDT insecticides

**Study Sites**	**Bendiocarb**			**DDT**			**Pirimiphos-Methyl**		

	**Number Assayed 342)**	**Mortality N (%)**	**Status**	**Number Assayed (344)**	**Mortality N (%)**	**Status**	**Number Assayed (324)**	**Mortality N (%)**	**Status**
**Kalorgu**	46	40 (87)	R	45	16 (35.6)	R	40	38 (95)	R
**Ture**	41	31 (75.6)	R	46	13 (28.3)	R	40	34 (85)	R
**Bambam**	41	41 (100)	**S**	42	7 (16.7)	R	43	31 (72.1)	R
**Pantami**	43	43 (100)	**S**	40	10 (25)	R	40	27 (67.5)	R
**Pokolin**	42	12 (28.6)	R	41	0 (0)	R	40	26 (65)	R
**Zazzagawa**	44	1 (2.2)	R	44	8 (18.2)	R	41	27 (65.9)	R
**Filiya**	44	29 (65.9)	R	44	10 (22.7)	R	40	30 (75)	R
**Diga**	40	24 (60)	R	42	16 (38.1)	R	40	32 (80)	R

N= number of mortality, R= Resistant, S= Susceptible, Numbers in brackets represent percentages No mortality was observed in the control experiment set up for the insecticides in each study site

A GLM procedure to compare mortality in *An. gambiae* s.l. populations exposed to two different insecticides showed no significant difference between mortality of *An. gambiae* populations exposed to lambdacyhalothrin and deltamethrin (t=−1.58906, P=0.1344). However, mortalities were significantly higher in both lambda cyhalothrin (t= 5.1234, P= 0.0002) and deltamethrin (t= 5.09965, P= 0.0002) exposed populations of *An. gambiae* s.l. mosquitoes compared to permethrin. Lambda cyhalothrin and deltamethrin insecticides produced the highest mortality in the population of *An. gambiae* in most of the study sites compared to permethrin. In comparing mortalities in *An. gambiae* populations exposed to two different classes of insecticides (organochlorine/ pyrethroids and bendiocarb/ organophosphate), mortality rate was significantly higher in *An. gambiae* populations exposed to deltamethrin (t= −0.55542, P=0.587372) and lamdacyhalothrin (t=5.07487, P= 0.000169) compared to DDT. In contrast, there was no significant difference between mortalities observed from *An. gambiae* s.l. populations exposed to DDT and permethrin (t= −0.55542, P= 0.587372). Similarly, a comparison of differences in mortality rates between *An. gambiae* s.l*.* populations exposed to bendiocarb (carbamate) and pirimiphos-methyl (organophosphate) in all the study sites were also not significant (t= −0.84482, P= 0.412419).

### Species composition of members of *Anopheles gambiae* s.l.

Out of the 2019 *An. gambiae* s.l. mosquitoes subjected to the bioassay, three hundred *An. gambiae* s.l. mosquitoes selected across the study communities were assayed to detect sibling species. *Anopheles gambiae* (formerly referred to as ‘S’ molecular form) and *An. coluzzii* (formerly referred to as ‘M’ form), *An. arabiensis* and hybrid (*An. gambiae*/ *An. coluzzii*) were all found across the study communities (Table 3). *Anopheles gambiae* (formerly S-form) was the significantly dominant species across the study communities constituting 177 (45%) of the total samples analyzed (Table 3). *Anopheles arabiensis* accounted for 64 (16.3 %) of the total samples scored in the assay. On the other hand *An. coluzzii* (M-form) constituted 34 (8.7%). The mosquitoes unaccounted for constituted about 40 (10.2%) and these were samples either misidentified samples or samples with poorly preserved DNA (Table 3).

**Table 3.** Distribution and composition of members of *Anopheles gambiae* s.l. in the study areas

**Study Sites**	**Number Assayed (N)**	**Species composition**				

		***A. arabiensis***	***An. gambiae* (S-form)**	***An. coluzzii* (M-form)**	**Hybrid**	**No amplification (NA)**
**Kalorgu**	70	13(18.6)	6(8.6)	2(2.9)	41(58.6)	8(11.4)
**Ture**	52	13(18.6)	22(42.3)	2(3.8)	10(19.2)	5(9.6)
**Bambam**	21	6(28.6)	10(47.6)	0(0)	3(14.3)	2(9.5)
**Pantami**	63	7(11.1)	34(54)	6(9.5)	7(11.1)	9(14.2)
**Pokolin**	58	7(12.1)	34(58.6)	7(12.1)	5(8.6)	5(8.6)
**Zazzagawa**	74	8(10.8)	49(66.2)	11(14.9)	6(8.1)	0(0)
**Filiya**	15	1(6.6)	10(66.7)	3(20)	0(0)	1(6.6)
**Diga**	40	9(22.5)	12(30)	3(7.5)	6(15)	10(25)
**Total**	393	64(16.3)	177(45)	34(8.7)	78(19.8)	40(10.2)

N= Total number of *An. gambiae* s.l., NA= No amplification, Numbers in brackets represent percentage

Outside the 40 (10.2%) samples not successfully amplified, analysis of variance to test difference in species composition of members of the *An. gambiae* s.l. was significant (P= 0.011369). Results showed that *An. gambiae* was significantly higher compared to *An. coluzzii* (P= 0.006433.) and *A. arabiensis* (P= 0.024755). There was no significant difference between *An. gambiae* and the hybrid species (P= 0.103996) (Fig. 2).

## Discussion

Resistance of *An. gambiae* s.l. mosquitoes to all the four major classes of insecticides were observed in all the study communities in southern Gombe. This widespread observation of resistance in the major vector *An. gambiae* s.l. and factors responsible for this upsurge in these communities is not known and should be investigated. However, the absence of continuous monitoring data in these communities may have denied vector control managers the opportunity to have detected this trend earlier.

Resistance of *An. gambiae* s.l. to several classes of insecticides is not entirely new in Nigeria (4, 17). Permethrin, bendiocarb and DDT resistance were have been reported in *An. coluzzii* mosquitoes in Kano, Northern Nigeria (11). Summary of insecticide resistance cases and number of countries reporting this have constantly been on the rise (2). However, a consideration of factors (usage of ITN, IRS, agricultural pesticides) previously reported selecting for insecticide resistance (17, 18) provided a reasonable clue of what could be the driver of insecticide resistance in Southern Gombe.

Utilization of insecticide nets by persons in Gombe is 34%, which is just below the national average of 37% (1). Gombe state ranked 8th out of the 38 states with the highest percentage of households with at least one ITN (1). There is a significant usage of Insecticide treated nets in Gombe state in Nigeria hence, these insecticide based tools may have contributed to the insecticide pressure in selecting for resistance in the *Anopheles gambiae* population. On the other hand, the use of IRS may not have added significantly to this present scale of insecticide resistance because coverage of IRS in all the states in the 6 geopolitical zones of Nigeria is just 2.5% (1). As such, there seem to be some other major contributors to the multiple insecticide pressure occasioned in these localities.

A literature search for recent update on pesticide utilization in Gombe state yielded a positive result on what may have been the source of these insecticide pressures. WAAP (19) reported the widespread cultivation of horticultural crops on which considerable amount of pesticides of different classes are used in Gombe state. Hence, by inference, agricultural use of insecticides therefore may have contributed to the current modification of mosquito susceptibility to the pesticides in Southern Gombe. The status of pyrethroid resistance status of *An. gambiae* s.l. population in all the study sites is worrisome considering that the major malaria vector control tools in Nigeria rely on this class of insecticides (20). However, the disparities in mortality rates expressed by the Anopheles population to each of the pyrethroid insecticides indicate that rate of resistance development vary with different insecticides in each location and population. For example, while higher mortality rates (51.2–100%) to lamdacyhalothrin was observed in the Anopheles. populations at all the study sites, lower mortality rates were observed in populations exposed to permethrin (0–46.3%) and deltamethrin (39–70%).

This variation is an important indication of the need to introduce insecticide resistance management strategies to slow down the rate at which these mosquitoes develop resistance to the pyrethroids insecticides. This will invariably help in prolonging the shelf life of the few available classes of insecticides on which ITNs and IRS rely upon. Recent report from an agricultural setting in Jigawa state: another northern state in Nigeria showed multiple resistances to a pyrethroid, carbamate and an organochlorine (10). Similar evidences of full susceptibility to lambda-cyhalothrin as reported in Bambam study community have also been reported in Kenya (21), Sudan (22), Tanzania (23) and Nigeria (24). In observing the pattern of resistance in the vectors, full susceptibility to bendiocarb was observed in, Bambam and Pantami which are both neighbhouring communities.

This suggests the focal nature of the insecticide resistance status of *Anopheles* populations in each of these communities. While bendiocarb resistance was observed in Anopheles population in 6 out of the 8 communities, resistance to pirimiphos-methyl was observed in the *An. gambiae* populations from all the communities tested. Resistance to pirimiphos-methyl (an organophosphate) in Gombe is of great concern to vector control manager because it is not currently used in any of the current vector control tools. However, the widespread resistance in *Anopheles* population in 8 communities to pirimiphos-methyl (organ-ophosphate) and the co-resistance to bendiocarb (a carbamate with same target site with organophosphate) point towards other sources as these have not been used for vector control. In contrast, findings from Auyo, a community in Jigawa, Nigeria, Kouande and Tanguieta in Atacora communities, in Republic of Benin, the *An. gambiae* populations were susceptible to pirimiphos-methyl (10, 25). In this study, all the *Anopheles* populations were resistant to deltamethrin exposure in all the study communities. Similar findings of deltamethrin resistance have been reported in Benin, West Africa (26, 27).

Contrary to the findings of this study, in Khartoum city of Sudan, high susceptibility to deltamethrin was reported (22). In this study, similar lower mortality of *Anopheles* mosquitoes from all the study communities to permethrin have been reported severally in Nigeria: Oyo, Lagos, Niger and Bauchi states (4, 24). Similar evidence of resistance to permethrin have also been reported in Atacora, Benin (27). Evidence of DDT resistance was high in this present study and this was not surprising considering that both pyrethroids and DDT (organochlorine) share the same target site. High resistance to one class may have led to cross-resistance in the other class. Insignificant differences in the mortality rates from permethrin and DDT mortality in the Anopheles population also showed similar patterns of resistance.

A molecular analysis would have provided additional information on probable existence of similar resistance mechanisms in the populations; however, logistics was a major limitation to this study. Several studies in Nigeria have reported high levels of permethrin-DDT resistance in *An. gambiae* and *An. coluzzii* in Nigeria (4, 6, 8 and 24). Evidence of resistance to pirimiphos-methyl (mortality rate between 65–95%) was observed in the *An. gambiae* populations in all the study communities. In comparing mortalities in *An. gambiae* populations exposed to two different classes of insecticides (organochlorine/ pyrethroids and bendiocarb/ organophosphate), showed there is significant differences in mortality rates of populations exposed to deltamethrin and lambda cyhalothrin (class II pyrethroids). Deltamethrin and lambda cyhalothrin are still effective compared with permethrin (a class I pyrethroids). Similarly, a comparison of differences in mortality rates between *An. gambiae* s.l*.* populations exposed to bendiocarb (carbamate) and Pirimiphos-methyl (organophosphate) in all the study sites were also not significant suggesting possible shared resistance mechanisms. This however requires laboratory validation.

Identification of the *Anopheles* species collected in this study showed the predominance of *An. gambiae* s.l. as the major malaria vectors in all the study communities. The *An. gambiae* complex includes sibling species that are the most important vectors of malaria in subsaharan Africa (28). Of these species *An. gambiae* (formerly S-form), *An. coluzzii* (formerly M-form) *A*. *arabiensis* and hybrid species (*An. gambiae*/ *An. coluzzii)* were found to be sympatric in all the study communities. In this study, *An. gambiae* (formerly S form) was the predominant species over *An. arabiensis*, *An. coluzzii* and the hybrid species identified. The sympatric occurrence of *An. gambiae*, *An. coluzzii* and *An. arabiensis* species have been reported in North–Central Nigeria (29).

While this study reported, a higher proportion (19.8%) of *An. gambiae*/*An. coluzzii* hybrids than any previous report in Nigeria (6, 12, 30). Information on the role of these hybrids and their contribution to malaria transmission is currently unavailable. In this study, *An. gambiae* s.s (formerly S form) was found to be the most predominant species. In contrast, this species was either found to be entirely absent or in insignificant proportions where the hybrid occurred with predominant *An. coluzzii* (6, 10, 12, and 30). This raises questions on the fitness status of these hybrids compared to the disappearing *An. gambiae* when exposed to insecticides. The preponderance of *An. gambiae* (formerly S form) over *An. coluzzii* species has always been attributed to ecological preference for drier areas of the savannah regions or areas with temporary breeding habitats. The species composition outcome of this study is in agreement with previous nationwide mapping of the distribution of members of the *An. gambiae* complex in the Sudan savannah where the study communities are located (31, 32).

## Conclusion

Monitoring insecticide resistance is an integral part of measures against malaria vectors and the resistance management strategies depends on the knowledge of the composition of malaria vectors in an area as well as their resistance profiles. *Anopheles gambiae*, *An. coluzzii*, *An. arabiensis*, and the hybrid species identified in all the study communities in Southern Gombe were resistant to all the four classes of insecticide approved for vector control. There is an urgent need for the implementation of insecticide resistance management strategies in these localities. Further studies must also be implemented to assess the operative resistance mechanisms in order to inform the choice of alternative vector control interventions to be considered.

## References

[1] National Population Commission (Nigeria). Federal Ministry of Health of Nigeria National Bureau of Statistics (Nigeria), and Institute for International Programs at Johns Hopkins Bloomberg School of Public Health A verbal/ social autopsy study to improve estimates of the causes and determinants of neonatal and child mortality in Nigeria (2014) Abuja, Nigeria, and Baltimore, Maryland, USA.

[2] World Health Organization (2012) World Malaria Report 2012.

[3] Oduola AO Olojede JB Ashiegbu CO Adeogun AO Otubanjo OA Awolola TS (2010) High Level of DDT Resistance in the Malaria Mosquito: *Anopheles gambiae* s.l. from Rural, Semi Urban and Urban Communities in Nigeria. J Rural and Trop Pub Health. 9: 114–120.

[4] Ibrahim KT Popoola KO Adewuyi OR Adeogun AO Oricha AK (2013) Susceptibility of *Anopheles gambiae* sensu lato to permethrin, deltamethrin and bendiocarb in Ibadan city, southwest Nigeria. Curr Res J Bio Sci. 5(2): 42–44.

[5] Okorie PN Ademowo OG Irving H Kelly-Hope LA Wondji CS (2015) Insecticide Susceptibility of *Anopheles coluzzii* and *Anopheles gambiae* Mosquitoes in Ibadan, Southwest Nigeria. Med Vet Entomol. 29: 44–50. 25417803 10.1111/mve.12089 PMC4319996

[6] Djouaka RJ Atoyebi SM Tchigossou GM Riveron JM Irving H Akoton R Kusimo MO Bakare AA Wondji CS (2016) Evidence of a multiple insecticide resistance in the malaria vector *Anopheles funestus* in South West Nigeria. Malar J. 15: 565. 27876039 10.1186/s12936-016-1615-9 PMC5120565

[7] Nwankwo EN Okorie PN Acha CT Okonkwo OE Nwangwu UC Ezihe EK (2017) Insecticide resistance in *Anopheles gambiae s.l*. mosquitoes in Awka, Anambra State, Southeast Nigeria. J Mosq Res. 7(5): 32–37.

[8] Adeogun AO Popoola KO Oduola AO Olakiigbe AK Awolola TS (2017) High level of DDT resistance and reduced susceptibility to deltamethrin in *Anopheles gambiae*, *Anopheles coluzzi*, and *Anopheles arabiensis* from Urban Communities in Oyo State, South-West Nigeria. J Mosq Res. 7(16): 125–133.

[9] Ibrahim SS Yayo AM Tukur Z Irving H Wondji CS (2014) High frequency of kdr L1014F is associated with pyre-throid resistance in Anopheles coluzzii in Sudan savannah of northern Nigeria. BMC Infectious Diseases. 14: 441. 25127882 10.1186/1471-2334-14-441 PMC4147187

[10] Alhassan AJ Sule MS Dan Gambo MA Yayo AM Safiyanu M Sulaiman D (2015) Detoxification Enzymes Activities in DDT and Bendiocarb resistant and susceptible malarial vector (*Anopheles gambiae*) breed in Auyo residential and irrigation sites, northwest Nigeria European Sci Journal. 11: 9.

[11] Abdu HU Manu YA Deeni YY (2017) Susceptibility status of *Anopheles gambiae* complex to insecticides commonly used for malaria control in Northern Nigeria. Int J Scientific Tech Res. 6(6): 47–54.

[12] Gillies M De Meillon B (1968) The Anophelinae of Africa South of the Sahara (Ethiopian zoogeographical region). Pub South Afr Inst for Med Res. 54: 343.

[13] Gillies MT Coetzee M (1987) A Supplement to the Anophelinae of Africa South of the Sahara. A Pub South Afr Inst for Med Res. 55: 33–81.

[14] Scott JA Brogon WG Collins FH (1993) Identification of Single Specimen of the *Anopheles* Complex by Polymerase Chain Reaction. Am J Trop Med Hyg. 49: 520–529. 8214283 10.4269/ajtmh.1993.49.520

[15] Fanello C Santolamazza F Della Torre A (2002) Simultaneous identification of species and molecular forms of the *Anopheles gambiae* complex by PCRRFLP. Med Vet Entomol. 16(4): 461–464. 12510902 10.1046/j.1365-2915.2002.00393.x

[16] WHO (2013) Test procedures for insecticide resistance monitoring in malaria vector mosquitoes. Geneva: World Health Organization; 2013.

[17] Abuelmaali SA Elaagip AH Basheer MA Frah EA Ahmed FTA Elhaj HFA Seidahmed OME David Weetman D Hamid MMA (2013) Correction: Impacts of Agricultural Practices on Insecticide Resistance in the Malaria Vector Anopheles arabiensis in Khartoum State, Sudan. PLoS One. 8(12): 10.137. 10.1371/journal.pone.0080549 PMC3832379 24260414

[18] Trape JF Tall A Diagne N Ndiath O Ly AB Faye J Dieye-Ba F Roucher C Bouganali C Badiane A Sarr FD Mazenot C Touré-Baldé A Raoult D Druilhe P Mercereau-Puijalon O Rogier C Sokhna C (2011) Malaria morbidity and pyrethroid resistance after the introduction of insecticide-treated bednets and artemisinin-based combination therapies: a longitudinal study. Lancet Infect Dis. 11(12): 925–932. 21856232 10.1016/S1473-3099(11)70194-3

[19] West Africa Agricultural Productivity Programme (2013) Report of a baseline study on status of use, registration and regulations of pesticides in Nigeria. Available at: https://www.slideshare.net/waapp-nigeria/final-version-of-baseline-studies-on-pestticide-use-submiited-to-waapp2013a1.

[20] World Health Organization (2016) World Malaria Report. Geneva: World Health Organization.

[21] Kamau L Agai D Matoke D Wachira L Gikandi G Vulule JM (2007) Status of Insecticide Susceptibility in *Anopheles* *gambiae* sensu lato and *Anopheles funestus* Mosquitoes from Western Kenya. J Insect Sci. 8: 11. 10.1673/031.008.1101 PMC3061582 20345290

[22] Seidahmed OME Abdelmajed MA Mustafa MS Mnzava AP (2012) Insecticide Susceptibility Status of the Malaria Vector *Anopheles arabiensis* in Khartoum City, Sudan: Differences between Urban and Periurban Areas. East Mediterr Health J. 18(7): 769–76. 22891527 10.26719/2012.18.7.776

[23] Protopopoff N Matowo J Malima R Kavishe R Kaaya R Wright A West PA Kleinschmidt I Kisinza W Mosha FW Rowland M (2013) High Level of Resistance in the Mosquito *Anopheles gambiae* to Pyrethroids Insecticides and Reduced Susceptibility to Bendiocarb in North-western Tanzania. Malar J. 12: 149. 23638757 10.1186/1475-2875-12-149 PMC3655935

[24] Umar A Kabir BGJ Amajoh CN Inyama PU Ordu DA Barde AA Misau AA Sambo ML Babuga U Kobi M Jabbdo MA (2014) Susceptibility Test of Female *Anopheles* Mosquitoes to Ten Insecticides for Indoor Residual Spraying (IRS) Baseline Data Collection in Northeastern Nigeria. J Entomol Nematology. 6(7): 98–103.

[25] Aïkpon R Sezonlin M Ossè R Akogbeto M (2014) Evidence of Multiple Mechanisms Providing Carbamate and Organophosphate Resistance in Field *A. gambiae* Population from Atacora in Benin. Parasit Vectors. 7: 568. 25443399 10.1186/s13071-014-0568-5 PMC4256734

[26] Aïzoun N Ossè R Azondekon R Alia Oussou O Gnanguenon V Aikpon R Padonou GG Akogbéto M (2013) Comparison of the Standard WHO Susceptibility Tests and the CDC Bottle Bio-assay for the Determination of Insecticide Susceptibility in Malaria Vectors and their Correlation with Biochemical and Molecular Biology Assays in Benin, West Africa. Parasit Vectors. 6: 147. 23688233 10.1186/1756-3305-6-147 PMC3669035

[27] Aïkpon R Ossè R Govoetchan R Sovi A Oké-Agbo F Akogbéto MC (2013) Entomological Baseline Data on Malaria Transmission and Susceptibility of *Anopheles gambiae* to Insecticides in Preparation for Indoor Residual Spraying (IRS) in Atacora, (Benin). J Parasitol Vector Biol. 1(1): 035–044.

[28] Coetzee M (2004) Distribution of the African malaria vectors of the *Anopheles gambiae* complex. Am J Trop Med Hyg. 70: 103–104. 14993617

[29] Oduola AO Adelaja OJ Aiyegbusi ZO Tola M Obembe A Ande AT Awolola S (2016) Dynamics of Anopheline vector species composition and reported malaria cases during rain and dry seasons in two selected communities of Kwara State. Nig J Parasitol. 37(2): 157–163.

[30] Abdu HU Spiers AJ Simona H Dauda MM Deeni YY (2017) Malaria vectors resistance to commonly used insecticides in the control of Malaria in Bichi, Northern Nigeria. Bayero J Pure and App Sci. 10(1): 1–6.

[31] Onyabe DY Conn JE (2001) The distribution of two major malaria vectors, *Anopheles gambiae* and Anopheles arabiensis, in Nigeria. Mem Inst Oswaldo Cruz. 96(8): 1081–1084. 11784926 10.1590/s0074-02762001000800009

[32] Awolola TS Oyewole IO Amajoh CN Idowu ET Ajayi MB Oduola A Manafa OU Ibrahim K Koekemoer LL (2005) Distribution of the molecular forms of *Anopheles gambiae* and pyrethroid knockdown resistance gene in Nigeria. Acta Trop. 95: 204–209. 16023989 10.1016/j.actatropica.2005.06.002

